# Proximal femur fracture detection on plain radiography via feature pyramid networks

**DOI:** 10.1038/s41598-024-63001-2

**Published:** 2024-05-27

**Authors:** İlkay Yıldız Potter, Diana Yeritsyan, Sarah Mahar, Nadim Kheir, Aidin Vaziri, Melissa Putman, Edward K. Rodriguez, Jim Wu, Ara Nazarian, Ashkan Vaziri

**Affiliations:** 1 BioSensics, LLC, 57 Chapel Street, Newton, MA 02458 USA; 2 Carl J. Shapiro Department of Orthopaedic Surgery, Beth Israel Deaconess Medical Center (BIDMC) and Harvard Medical School, 330 Brookline Avenue, Stoneman 10, Boston, MA 02215 USA; 3 Musculoskeletal Translational Innovation Initiative, Beth Israel Deaconess Medical Center and Harvard Medical School, 330 Brookline Avenue RN123, Boston, MA 02215 USA; 4 Division of Endocrinology, Massachusetts General Hospital and Harvard Medical School, 55 Fruit Street, Boston, MA 02114 USA; 5 Department of Radiology, Massachusetts General Brigham (MGB) and Harvard Medical School, 75 Francis Street, Boston, MA 02215 USA; 6 Department of Orthopaedic Surgery, Yerevan State University, Yerevan, Armenia

**Keywords:** Plain radiography, Hip, Proximal femur, Fracture, Deep learning, Radiography, Predictive markers, Bone

## Abstract

Hip fractures exceed 250,000 cases annually in the United States, with the worldwide incidence projected to increase by 240–310% by 2050. Hip fractures are predominantly diagnosed by radiologist review of radiographs. In this study, we developed a deep learning model by extending the VarifocalNet Feature Pyramid Network (FPN) for detection and localization of proximal femur fractures from plain radiography with clinically relevant metrics. We used a dataset of 823 hip radiographs of 150 subjects with proximal femur fractures and 362 controls to develop and evaluate the deep learning model. Our model attained 0.94 specificity and 0.95 sensitivity in fracture detection over the diverse imaging dataset. We compared the performance of our model against five benchmark FPN models, demonstrating 6–14% sensitivity and 1–9% accuracy improvement. In addition, we demonstrated that our model outperforms a state-of-the-art transformer model based on DINO network by 17% sensitivity and 5% accuracy, while taking half the time on average to process a radiograph. The developed model can aid radiologists and support on-premise integration with hospital cloud services to enable automatic, opportunistic screening for hip fractures.

## Introduction

Hip pain is a common reason for presentation to an emergency room. A traumatic event such as a fall associated with hip pain or a deformity points to a high suspicion of hip fracture and is often easily diagnosed with radiography. However, some fractures are not obvious and can constitute an occult, valgus impacted, or nondisplaced fracture that can be missed in initial radiographic assessments, often requiring additional imaging modalities such as CT or MRI. In the United States, hip fractures exceed 250,000 annually, with an incidence of 80 per 100,000 population^1–3^. Hip fracture incidence rates are known to increase exponentially with age in both women and men^4^, secondary to osteoporosis and osteoporosis. This is the most common metabolic disease worldwide, predominantly affecting the elderly population^5^ and characterized by decreased bone mineral density and loss of trabecular architecture^6^, bone microstructural deterioration, and increased fracture risk^7^. A total of 8.9 million fractures are caused by osteoporosis in the world annually, resulting in an osteoporotic fracture every 3 s^8^. Although it is not the most common type of fragility fracture, osteoporotic hip fractures are considered the most serious, with a mortality rate reaching 20–40% during the first year after fracture^9,10^. With rising global life expectancy, the incidence and prevalence of osteoporosis are also expected to increase. Accordingly, the number of men and women combined who will be above the threshold for a fracture is expected to almost double by 2040, with a prediction of 319 million cases^11,12^. In fact, by 2050, the worldwide incidence of hip fracture in women is projected to increase by 240% and 310% in men^8,13^, with approximately 1 in 2 women and 1 in 5 men over the age of 50 projected to suffer from a fracture in their remaining lifetime^14^.

Hip fractures are prevalently detected via abnormalities observed on plain radiography, patient history, and physical examination findings. Nevertheless, radiographical appearance on radiography is not always sufficient for final diagnosis due to highly variable patient parameters such as BMI, positioning, and image quality^15^. Up to 10% of at-risk patients are examined via further imaging, including computed tomography (CT) or magnetic resonance imaging (MRI) to limit misdiagnosis. However, less than a third of the further studied cases subsequently demonstrate hip fractures^2,16^. Additional advanced imaging studies face challenges such as high cost and limited availability at remote and non-urban healthcare facilities. At the same time, delayed diagnosis and unrecognized fractures increase the risk of mortality^2^ and the time and cost of hospitalization^17^.

Employing an accurate automated detection model for hip fractures on radiographs can aid experts in saving time and resources. As a result, automated tools using machine learning and deep learning models have been increasingly studied in the literature^18,19^. Many studies have employed deep learning models trained over thousands of annotated radiographs and demonstrated high accuracy for potential clinical deployment^20–32^. Nevertheless, these approaches lacked explicit localization of identified hip fractures. Providing the location of identified fractures allows the clinician to visualize and overread automated detection results to confirm the result or decide on further evaluation. Thus, several studies have focused on detecting and localizing hip fractures from radiographs via deep learning, albeit requiring multiple cascaded models^33–37^. Developing and evaluating such cascaded approaches is less computationally efficient than end-to-end one-stage detection and localization^38–40^ and potentially requires manual data cleaning between cascaded models to address error propagation^36^.

Recent works have proposed deep learning models for end-to-end detection and localization of hip fractures from radiographs^41,42^, particularly focusing on feature pyramid networks (FPNs)^43–47^. FPNs are convolutional neural networks combining features extracted at different scales and resolutions toward object detection predictions. They are tailored for medical imaging applications in which variability in resolution and anatomical structure sizes are long-lasting challenges^48^. Despite their success, FPNs have been typically evaluated for generic object detection metrics such as average precision^45–47^, thereby limiting validation with clinically relevant metrics and confidence intervals. In recent years, transformer models have also become integral to deep learning approaches in medical image analysis, including detection and classification^49^. For hip fracture analysis from plain radiography, transformer models have only been employed within multi-stage cascaded approaches^36^, leaving room for end-to-end detection and localization as in FPNs.

Motivated by these observations, this study aims to assess state-of-the-art deep learning models for object detection, including FPNs and transformers, on end-to-end proximal femur fracture detection and localization from plain radiography with clinically relevant metrics. We employed and extended the VarifocalNet FPN^50^, well-established for object detection in various domains. Using a retrospective dataset of 823 hip radiographs from 150 subjects with proximal femur fractures and 362 negative controls, VarifocalNet attained 0.94 specificity and 0.95 sensitivity, with up to 14% sensitivity and 9% accuracy improvement against five benchmark FPN models. Crucially, we took the first steps in evaluating a transformer model for our task, employing the state-of-the-art DINO network^51^. We established that for commonly-observed small-sample settings such as ours, FPNs remain state-of-the-art: VarifocalNet outperformed DINO by 17% sensitivity and 5% accuracy while taking half the time on average to process a radiograph.

## Methods

### Study design

The Institutional Review Board in the Beth Israel Deaconess Medical Center (BIDMC) at Harvard Medical School approved this retrospective study in compliance with the Health Information Portability and Accountability Act. All data was collected at the BIDMC Division of Musculoskeletal Imaging & Intervention. Informed consent was obtained from all individual participants included in the study. All methods were performed in accordance with relevant guidelines and regulations following the Declaration of Helsinki.

We collected retrospective frontal view plain radiographs of the hip from subjects who sustained a proximal femur fracture after 2004 using the PACS system. The proximal femur joins with the acetabulum of the pelvis to form the hip joint. Hip radiographs from non-fracture age- and gender-matched subjects were used as controls. Fractures outside the proximal femur, such as the acetabulum, were also considered negative for proximal femur fracture analysis. Exclusion criteria included subjects with pathological fractures from pre-existing pathological diseases other than osteoporosis (history of bone cancer, infection, or cysts) and lateral view radiographs. Identified scans were exported, de-identified, and assigned a unique identifier before analysis. The resulting dataset included 440 hip radiographs from 122 subjects with proximal femur fractures and 194 hip radiographs from 194 controls without proximal femur fractures. To balance the number of fracture and control scans, we augmented this dataset with publicly available hip radiographs collected from 28 fracture subjects and 161 controls^52^. The final dataset comprised 468 hip radiographs from 150 subjects with proximal femur fractures and 355 hip radiographs from 355 negative controls without proximal femur fractures.

Table 1 shows the subject-by-subject distribution of gender, BMI, and race categories for fracture and control subjects. Supplementary Table S.1 shows the scan-by-scan distribution of age and gender categories for fracture and control subjects, as scans from the same fracture subject may be collected at different ages. Agreeing with the published reports on fracture incidence rates^14,53^, most fracture subjects were above the age of 50 for both genders, with more female subjects than male. The dataset was also diverse over the BMI categories, particularly for fracture subjects. While race distribution was more imbalanced, fracture incidences have been reported to exhibit higher rates over white populations compared to African American populations as in our dataset^53^. Supplementary Table S.2 shows the scan-by-scan distribution of imaging devices, exhibiting diverse representation over four different scanner device manufacturers. Included fracture cases also exhibited diversity over anatomical locations, including the greater trochanter (54%), intertrochanter (24%), femoral neck (20%), and femoral head (2%), as well as degree of displacement, including non-displaced and mild displacement cases. Agreeing with the literature, the most common fracture location was the greater trochanter^54^, and the rarest location was the femoral head^55^.

**Table 1:** Subject level demographics distribution.

	Fracture (n = 150, ages 26–100)	Control (n = 355, ages 18–97)
Gender, n (%)		
Female	87 (58%)	99 (27%)
Male	35 (23%)	95 (26%)
Unknown	28 (19%)	161 (47%)
BMI, n (%)		
Underweight (< 19)	11 (7%)	7 (3%)
Healthy weight (19–25)	31 (21%)	52 (14%)
Overweight (25–30)	14 (9%)	32 (10%)
Obese (> 30)	11 (7%)	27 (8%)
Unknown	83 (56%)	237 (65%)
Race, n (%)		
White	109 (73%)	152 (42%)
Black or African American	10 (7%)	19 (6%)
Asian	1 (1%)	1 (1%)
Hispanic	0	6 (2%)
Unknown	30 (19%)	177 (49%)

We also included the scan level age, gender proximal femur fracture presence and imaging device distribution in Supplementary Tables S.1 and S.2.

### Data annotation and partitioning

To perform fracture localization, a radiologist with clinical experience in musculoskeletal radiography manually annotated each confirmed fracture radiograph by drawing a bounding box that fully contained each visible fracture region using the PhotoPad Image Editor (NCH Software)^56^.

We partitioned our dataset into stratified training and test sets, keeping a uniform ratio of positive (with proximal femur fracture) and negative (without proximal femur fracture) subjects in each set. 10% of the subjects were held out for testing, and 90% were used for training. Data partitioning was based on subjects rather than scans, ensuring subjects included in training were not included in testing.

### Automated proximal femur fracture and localization via VarifocalNet

#### VarifocalNet architecture

We employed and extended the VarifocalNet feature pyramid network (FPN)^50^, motivated by the recent influx of FPN models for end-to-end detection and localization of hip fractures from radiographs^43–47^. VarifocalNet FPN was selected for its state-of-the-art prediction performance in detecting common objects, outperforming twenty-five object detection baselines^50^. In our application, VarifocalNet received a plain radiograph of the hip and made the following predictions: (i) rectangular bounding boxes circumscribing candidate fracture regions and (ii) a confidence score in the range 0–1 associated with each detected box. The confidence score governed the likelihood of fracture existence and was thresholded in post-processing stages to detect a fracture. We explain the details of VarifocalNet architecture and our approach below.

An FPN receives a 2D image of any size and begins with extracting a hierarchy of features at multiple scales via a base neural network^57^. The base network comprises a sequence of stages, each containing convolutional and residual layers. The activation output of each stage's last residual layer is part of the feature pyramid. Base network features are complemented by upsampling to extract higher-resolution features, merged with lower-resolution base network features of the same size to form a multi-scale feature pyramid. Feature extraction and merging at different resolutions tailors FPNs for medical imaging applications in which variability in resolution and anatomical structure sizes are long-lasting challenges^48^. We used the ResNeXt-101 architecture^58^ for the base network and five feature pyramid levels, following the recent literature on FPNs^47,50^. Features extracted by the feature pyramid were used for (i) detecting objects of interest via bounding boxes circumscribing object locations on the input image, and (ii) predicting a confidence score for each detected box.

To predict bounding boxes, VarifocalNet maps each pixel location at each feature pyramid level back to the original input scale by multiplying and shifting the pixel coordinates by the total stride before the current pyramid level^50^. For each pixel location, four scalars representing the distance to the object bounding box's left, top, right, and bottom corners were predicted. Neighboring locations around the current pixel location were selected and mapped back to the feature pyramid level to incorporate nearby contextual information. Formally, for a pixel with coordinates *x* and *y* along the width and height of the image, respectively, a bounding box was first predicted via a convolutional block. The distances from (*x, y*) to the left, top, right, and bottom corners of the bounding box were denoted by *l, t, r,* and *b*, respectively. To incorporate nearby contextual information, nine neighboring pixels with coordinates *(x, y), (xl, y), (x, y − t), (x* + *r, y), (x, y* + *b), (x − l, y–t), (x* + *l, y − t), (x − l, y* + *b)* and *(x* + *r, y* + *b)* were selected and mapped back to the feature pyramid level. Bounding box predictions were then refined by learning and incorporating residual improvement factors. In particular, four distance scaling factors (*∆l, ∆t, ∆r, ∆b)* were predicted via deformable convolution^59^ based on the features of neighboring pixels, where the relative offsets of neighboring pixels to (*x, y*) served as the offsets to the deformable convolution. The refined bounding box was then represented by $(l{\prime}, t{\prime}, r{\prime},$ $b{\prime})$ = *(∆l* × *l, ∆t* × *t, ∆r* × *r, ∆b* × *b*). Confidence score prediction followed the same steps as bounding box prediction except for the last layer, where the output was a scalar score *p* for each location (*x, y*), rather than the four distance factors.

**Figure 1:**
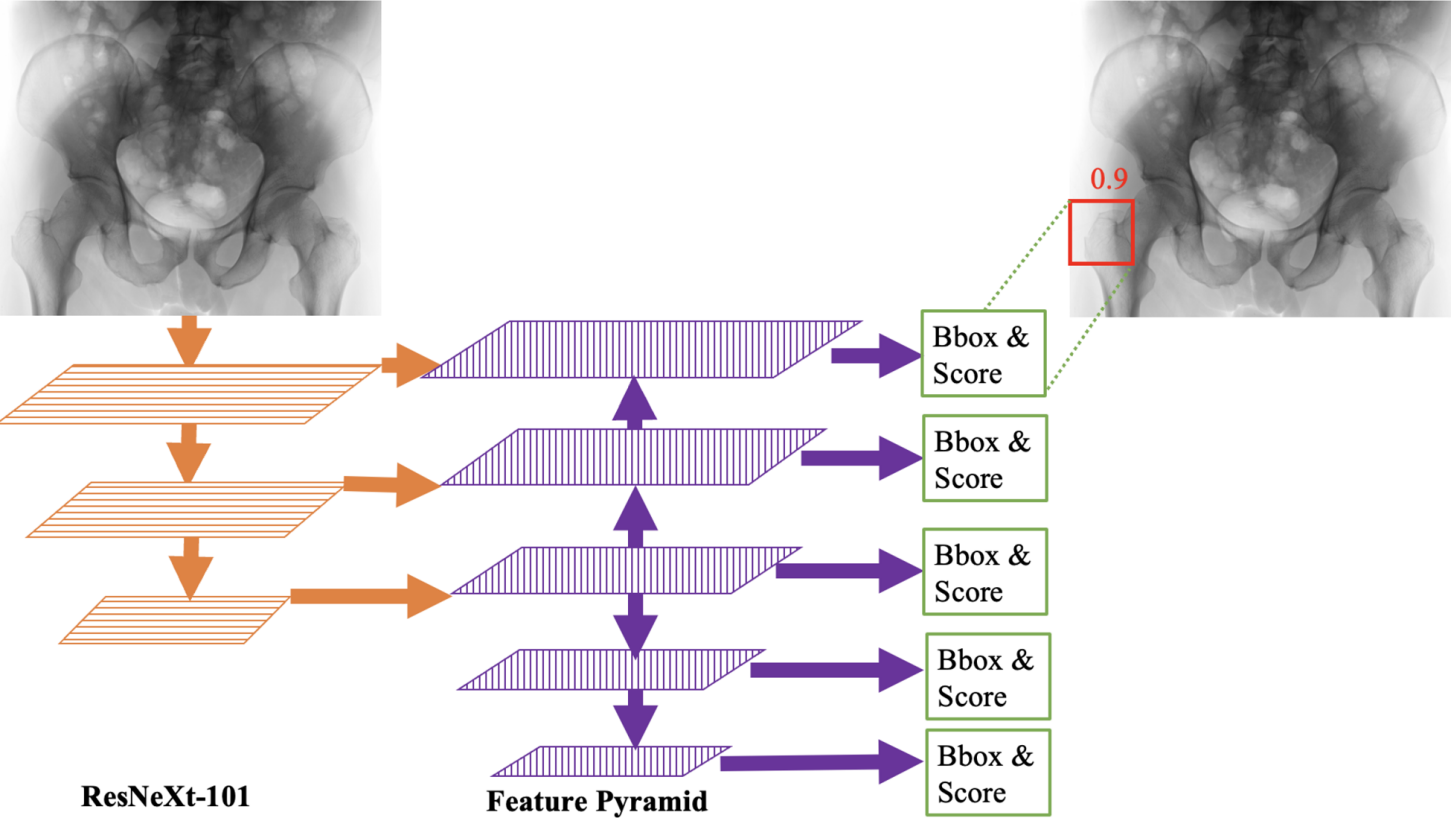
VarifocalNet architecture. The ResNeXt-101 base neural network comprises a sequence of stages, each containing convolutional and residual layers. These stages extract hierarchical multi-resolution features, depicted by rectangles with horizontal lines. ResNeXt-101 features are complemented by upsampling to extract higher-resolution features, depicted by rectangles with vertical lines. Higher and lower resolution features are merged and used for predicting fracture bounding boxes (bbox) and associated confidence scores in the range 0–1. We also included the detailed architecture breakdown for base neural network, feature pyramid, bounding box, and confidence score prediction stages in Supplementary Tables S.3–S.5.

Figure 1 summarizes the overall VarifocalNet architecture. We also included the detailed architecture breakdown for base neural network, feature pyramid, bounding box, and confidence score prediction stages in Supplementary Tables S.3–S.5.

#### Data preprocessing

We prepared each radiograph via contrast-limited adaptive histogram equalization (CLAHE) to enhance input radiographs, a typical technique in radiography-based fracture detection to reduce noise and improve image quality^60–62^. Each image was then normalized to the range 0–1 via min–max normalization, following the standard in deep learning literature for medical imaging to aid training stability^63,64^.

#### Training

Transfer learning was employed to accelerate training by initializing VarifocalNet parameters with weights pre-trained on a benchmark object detection dataset named COCO^65^. Following initialization, VarifocalNet was trained over the pairs of training scans and corresponding ground-truth fracture bounding boxes for 75 epochs via stochastic gradient descent with a momentum factor of 0.9 and batch size of 1^66^. The learning rate was initialized at $5\times$ 10^–3^ and divided by ten after every 25 epochs to aid training convergence^67^. To aid performance generalization, training scans were augmented by horizontal flipping and resizing, where image height was fixed at 1333 and width is varied between 512 and 800 by increments of 32. Moreover, initialized parameters of the first base network stage were not fine-tuned, while all trained parameters were regularized via weight decay with regularization level 10^–4^^68^. Initialization, optimization, data augmentation, and regularization techniques followed the standard in deep learning literature for object detection^50,51,57^.

The training objective comprised several components for optimizing bounding boxes and confidence scores. Fracture confidence scores were optimized by minimizing a weighted binary cross entropy loss to combat the imbalance between pixels pertaining to background vs. fractures^50^:

1 $$ - \frac{1}{\left| F \right|}\sum\limits_{i \in F} {q_{i} \left( {q_{i} log\left( {p_{i} } \right) + \left( {1 - q_{i} } \right)log\left( {1 - p_{i} } \right)} \right)} - \frac{1}{\left| F \right|}\sum\limits_{i \in B} {0.75 p_{i}^{2} log\left( {1 - p_{i} } \right)} , $$

where index *i* denotes a pixel location, *F* comprises the indices of foreground pixel locations coinciding with ground-truth fracture boxes, *B* comprises the indices of background pixel locations, *q* denotes the target confidence score*, p* denotes the predicted confidence score and $\left| F \right|$ denotes the number of foreground pixel locations. To capture the coupling between bounding box and confidence score predictions, the target score *q* took on the value of Intersection over Union (IOU)^69^ between ground-truth and predicted bounding boxes for foreground pixel locations and the value 0 otherwise. In doing so, detections outside ground-truth fracture boxes were assigned lower weights, while high-confidence detections overlapping with ground-truth boxes were assigned higher weights.

Fracture bounding box predictions were optimized by minimizing a generalized IOU (GIOU) objective^70^, governed by the negative of the proximity between a ground-truth fracture box and the corresponding detected box:

2 $${\sum }_{i \in F}\begin{array}{c}-\frac{1.5}{\left|F\right|} {q}_{i }GIOU\left(\left[{l}_{i} ,{t}_{i} ,{r}_{i} ,{b}_{i}\right], \left[{{l}_{i}}^{*},{{t}_{i}}^{*},{{r}_{i}}^{*},{{b}_{i}}^{*}\right]\right)\\ -\frac{2}{\left|F\right|} {q}_{i }GIOU\left(\left[{l}_{i}^{\prime}, {t}_{i}^{\prime}, {r}_{i}^{\prime}, {b}_{i}^{\prime}\right], \left[{{l}_{i}}^{*},{{t}_{i}}^{*},{{r}_{i}}^{*},{{b}_{i}}^{*}\right]\right),\end{array}$$

where * denotes the distance factors for a ground-truth fracture box. VarifocalNet was trained by minimizing the sum of (1) and (2), where the weighting coefficients 0.75, 1.5 and 2 followed Zhang et al.^50^.

#### Inference and evaluation metrics

We applied the trained fracture detection model on each scan in the test set to record bounding box detections and their confidence scores. We represented each scan with the detection corresponding to the maximum confidence score in the scan, to be thresholded for fracture detection. We determined the fracture detection threshold as the score that maximized the geometric mean of sensitivity and specificity^71^. In the clinical care environment aided by this binary prediction, experts are expected to review the positive-flagged scans and decide on fracture presence. Thus, the focus of our model was not to miss positive scans by focusing on one high-confidence detection for each scan.

Fracture detection performance was assessed via several clinically relevant evaluation metrics. Using the confidence scores before thresholding, the Area Under the Receiver Operating Characteristic Curve (AUC) was computed. After thresholding for binary classification of each scan as positive or negative for proximal femur fracture, sensitivity, specificity, accuracy, and positive and negative predictive values were computed as follows:

3 $$\text{Sensitivity}=\frac{\#\, of\, positive\, predictions}{\#\, of\, ground-truth\, positives}=\frac{\#\, of\, true\, positives\, (TP)}{TP + \#\, of\, false\, negatives\, (FN)},$$

4 $$\text{Specificity }=\frac{\#\, of\, negative\, predictions}{\#\, of\, ground-truth\, negatives}=\frac{\#\, of\, true\, negatives\, (TN)}{TN + \#\, of\, false\, positives\, (FP)},$$

5 $$\text{Accuracy}=\frac{TP+TN}{TP+TN+FN+FP},$$

6 $$\text{Positive Predictive Value }\left(\text{PPV}\right)=\frac{TP}{TP+FP},$$

7 $$\text{Negative Predictive Value }\left(\text{NPV}\right)=\frac{TN}{TN+FN}.$$

The benchmark IOU metric^69^ was used to assess fracture localization performance, governed by the overlap percentage between a ground-truth fracture box and the corresponding detected box. IOU was computed over the true positive scans, as these were the only scans with both ground-truth and detected fracture boxes after thresholding for fracture detection.

We reported each metric and its 95% confidence interval^72^. To assess the significance when comparing two metrics, we reported p-values for the two-sided Mann–Whitney nonparametric test^73^, as performance metrics do not follow a specific parametric distribution.

#### Competing methods

We evaluated VarifocalNet against five benchmark FPNs that have been tested for end-to-end hip fracture detection and localization from plain radiography: Faster-RCNN^44,74^, Cascade-RCNN^75^, RetinaNet^76^, Fully convolutional one-stage (FCOS)^77^ and Global Context networks (GCNet)^47^. Faster R-CNN and Cascade R-CNN involve region proposal networks to predict bounding box locations relative to pre-defined anchor boxes. RetinaNet incorporates focal loss to combat the imbalance between background vs. object locations. Similar to VarifocalNet, FCOS does not require anchor boxes and directly predicts bounding boxes and confidence scores for each pixel location on feature pyramids. GCNet combines region proposal networks with global context blocks to capture long-range dependencies over input images. Other FPNs from the literature on end-to-end hip fracture detection and localization from plain radiography included dilated convolutional feature pyramid network (DCFPN)^45^ and ParallelNet^46^, which were outperformed by the GCNet we implemented and compared with^47^. For fair comparison to VarifocalNet, all FPNs were implemented with ResNeXt-101 as their base neural network.

In addition to FPN benchmarks, we implemented the state-of-the-art DINO transformer network^51^ for end-to-end proximal femur fracture detection and localization from plain radiography. DINO uses a Swin transformer as the base neural network for feature extraction^78^ and a transformer encoder-decoder network for object detection and localization using Swin features. Transformer networks involve attention mechanisms that learn weighting coefficients over features to capture long-range dependencies^79^. For fair comparison to VarifocalNet, all base neural networks for FPNs and DINO were initialized with weights pre-trained on COCO and were implemented with the same preprocessing and inference procedures described in Sections "Data preprocessing" and "Inference and evaluation metrics".

Beyond end-to-end detection and localization approaches, we implemented two other state-of-the-art deep learning models commonly used for hip fracture detection. DenseNet^80^ employs dense connections by receiving features extracted by all preceding layers with identical feature shapes as inputs to each layer and has been used by a plethora of recent works^21,24,30–32^. We implemented the DenseNet-121 version following recent works^31,32^. EfficientNet was proposed to improve the efficiency of well-established convolutional neural networks by increasing architecture depth, resolution scaling and number of channels in intermediate layers to extract more fine-grained features^81^. It has been used by multiple related works^20,82^; we implemented the EfficientNet-B5 version following recent works^82^. Both networks were initialized with weights pre-trained over the benchmark image classification dataset ImageNet^83^ and implemented with the same preprocessing and inference procedures described in Sections "Data preprocessing" and "Inference and evaluation metrics".

### Ethics approval and informed consent

The Institutional Review Board in the Beth Israel Deaconess Medical Center (BIDMC) at Harvard Medical School approved this retrospective study in compliance with the Health Information Portability and Accountability Act. All data was collected at the BIDMC Division of Musculoskeletal Imaging and Intervention. Informed consent was obtained from all individual participants included in the study. All methods were performed in accordance with relevant guidelines and regulations following the Declaration of Helsinki.

## Results

Our goal in this study was to establish the state-of-the-art in deep learning models for end-to-end proximal femur fracture detection and localization from plain radiography with clinically relevant metrics. We present our relevant results below.

### Quantitative results

Table 2 visualizes the fracture detection and localization performance metrics for VarifocalNet against all competing methods. VarifocalNet attained high performance across all clinically relevant metrics, with 0.98 AUC, 0.94 specificity, 0.95 sensitivity, and 0.94 accuracy. In doing so, VarifocalNet outperformed all other FPN models by up to 6% AUC, 14% sensitivity, 9% accuracy, and 12% NPV, with p-values < 10^–4^. Moreover, VarifocalNet obtained the best balance between sensitivity and specificity.

**Table 2:** Comparison of VarifocalNet to competing methods.

Method		AUC	Specificity	Sensitivity	Accuracy	NPV	PPV	IOU
VarifocalNet	Metric	**0.98**	0.94	**0.95**	0.94	**0.94**	0.95	0.67
	CI ($\pm$)	0.03	0.06	0.05	0.06	0.06	0.05	0.09
DINO	Metric	0.91	**1.0**	0.78	0.89	0.81	**1.0**	**0.71**
	CI ($\pm$)	0.07	10^–3^	0.1	0.07	0.09	10^–3^	0.08
	p-value	10^–19^	10^–21^	10^–25^	10^–15^	10^–24^	10^–24^	10^–6^
FasterRCNN	Metric	0.95	0.97	0.89	0.93	0.89	0.97	0.67
	CI ($\pm$)	0.05	0.04	0.07	0.06	0.07	0.04	0.09
	p-value	10^–11^	10^–14^	10^–17^	0.03	10^–12^	10^–5^	0.03
CascadeRCNN	Metric	0.96	0.97	0.86	0.92	0.87	0.97	0.71
	CI ($\pm$)	0.05	0.04	0.08	0.06	0.08	0.04	0.08
	p-value	10^–6^	10^–8^	10^–22^	10^–9^	10^–18^	10^–5^	10^–4^
RetinaNet	Metric	0.96	0.91	0.89	0.9	0.89	0.92	0.69
	CI ($\pm$)	0.05	0.07	0.07	0.07	0.07	0.06	0.09
	p-value	10^–7^	10^–9^	10^–17^	10^–12^	10^–14^	10^–5^	10^–3^
FCOS	Metric	0.92	0.89	0.81	0.85	0.82	0.88	0.71
	CI ($\pm$)	0.07	0.07	0.09	0.08	0.09	0.08	0.08
	p-value	10^–22^	10^–13^	10^–25^	10^–23^	10^–23^	10^–20^	10^–8^
GCNet	Metric	0.96	0.97	0.84	0.9	0.85	0.97	0.67
	CI ($\pm$)	0.05	0.04	0.08	0.07	0.08	0.04	0.09
	p-value	10^–5^	10^–8^	10^–24^	10^–8^	10^–22^	10^–4^	0.03
DenseNet	Metric	0.97	0.97	0.95	**0.96**	0.94	0.97	N/A
	CI ($\pm$)	0.03	0.03	0.05	0.04	0.06	0.03	
	p-value	10^–6^	10^–5^	0.88	10^–4^	0.43	10^–6^	
EfficientNet	Metric	0.96	0.94	0.95	0.94	0.94	0.95	N/A
	CI ($\pm$)	0.04	0.05	0.05	0.05	0.05	0.05	
	p-value	10^–8^	0.57	0.88	0.6	0.43	0.09	

Fracture detection metrics include area under the receiver operating characteristic curve (AUC), sensitivity, specificity, accuracy, positive predictive value (PPV) and negative predictive value (NPV). Fracture localization is assessed by Intersection over Union (IOU). For each metric, the row with the highest value is written in bold. Below each metric, the 95% confidence interval (CI) is written. For each method other than the proposed VarifocalNet, the p-values (for the two-sided Mann–Whitney nonparametric test) for comparing the metrics to those of VarifocalNet are also written.

Crucially, VarifocalNet outperformed the DINO transformer network by 7% AUC, 17% sensitivity, 5% accuracy, and 13% NPV. DINO also attained the lowest AUC and largest imbalance between specificity and sensitivity among all methods. Our results confirmed that while transformer models have been widely employed for medical image analysis^49^, their performances on small-scale medical imaging datasets such as ours can vary substantially^84^. VarifocalNet not only outperformed DINO with clinically relevant metrics but also performed inference more efficiently: when evaluated on a Quadro RTX 6000 Graphical Processing Unit (GPU), VarifocalNet took 1.16 s on average to process each radiograph, while DINO took 2.13 s. Quantitative comparisons showed that for small-sample settings such as ours, FPNs remain state-of-the-art compared to transformer models.

Regarding fracture localization, all methods attained similar IOUs in the range of 0.67 to 0.71. As discussed in more detail below in Section "Qualitative results", VarifocalNet consistently localized fracture regions of interest correctly compared to the corresponding ground-truths, while detected box sizes and aspect ratios varied and lowered the average IOU.

In comparison to DenseNet and EfficientNet that only performed fracture detection, VarifocalNet attained similarly high detection performance with significantly better AUC, lower specificity, and equal sensitivity. Crucially, in doing so, VarifocalNet additionally provided the locations of identified fractures. Providing the location of identified fractures allows the clinician to visualize and overread automated detection results to confirm the result or decide on further evaluation.

We further analyzed VarifocalNet for gender subgroups: the average AUC was 0.99 for female subjects and 0.84 for male subjects. Agreeing with the literature on hip fractures^14,53^, our dataset comprised twice the number of female subjects than male subjects with proximal femur fractures, as summarized in Table 1. Thus, the trained model could generalize well over female subjects while remaining more limited in evaluations of male subjects.

### Qualitative results

**Figure 2:**
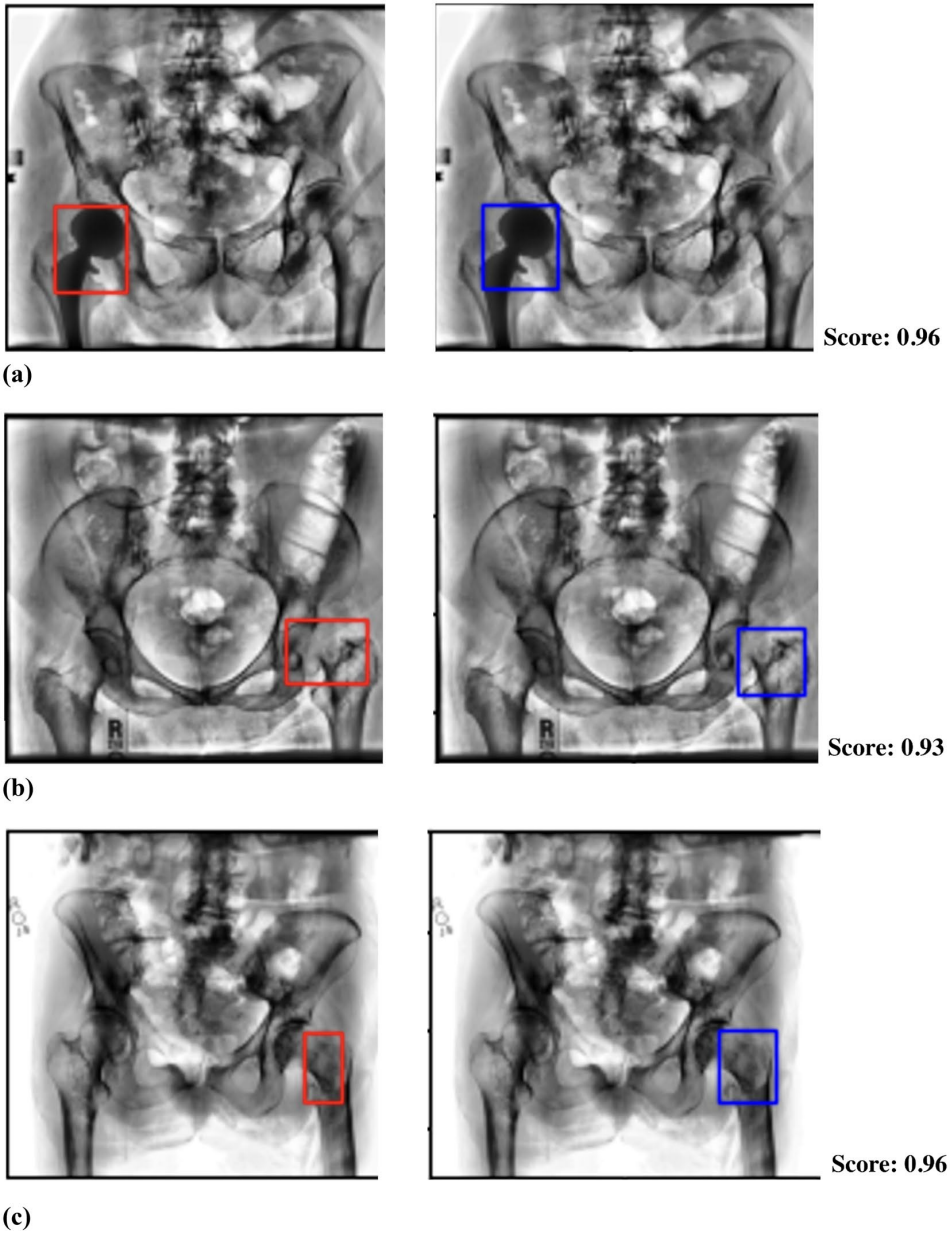
Example visualizations of ground-truth fracture bounding boxes (left) vs. predicted fracture bounding boxes by VarifocalNet (right). Images are radiographs preprocessed via CLAHE, as described in Section "Data preprocessing".

Figure 2 visualizes examples of ground-truth fracture bounding boxes vs. the corresponding predictions by VarifocalNet. VarifocalNet consistently localized fracture regions of interest correctly compared to the corresponding ground-truths, with particularly high confidence scores for scans with hip implants such as Fig. 2a. That said, detected box sizes and aspect ratios varied (c.f. Fig. 2b,c) and lowered the average IOU for all methods, as reported in Table 2. Overall, VarifocalNet prioritized highly accurate proximal femur fracture detection for clinical applications with expert review aided by localization, rather than the exact sizes of fractures.

**Figure 3:**
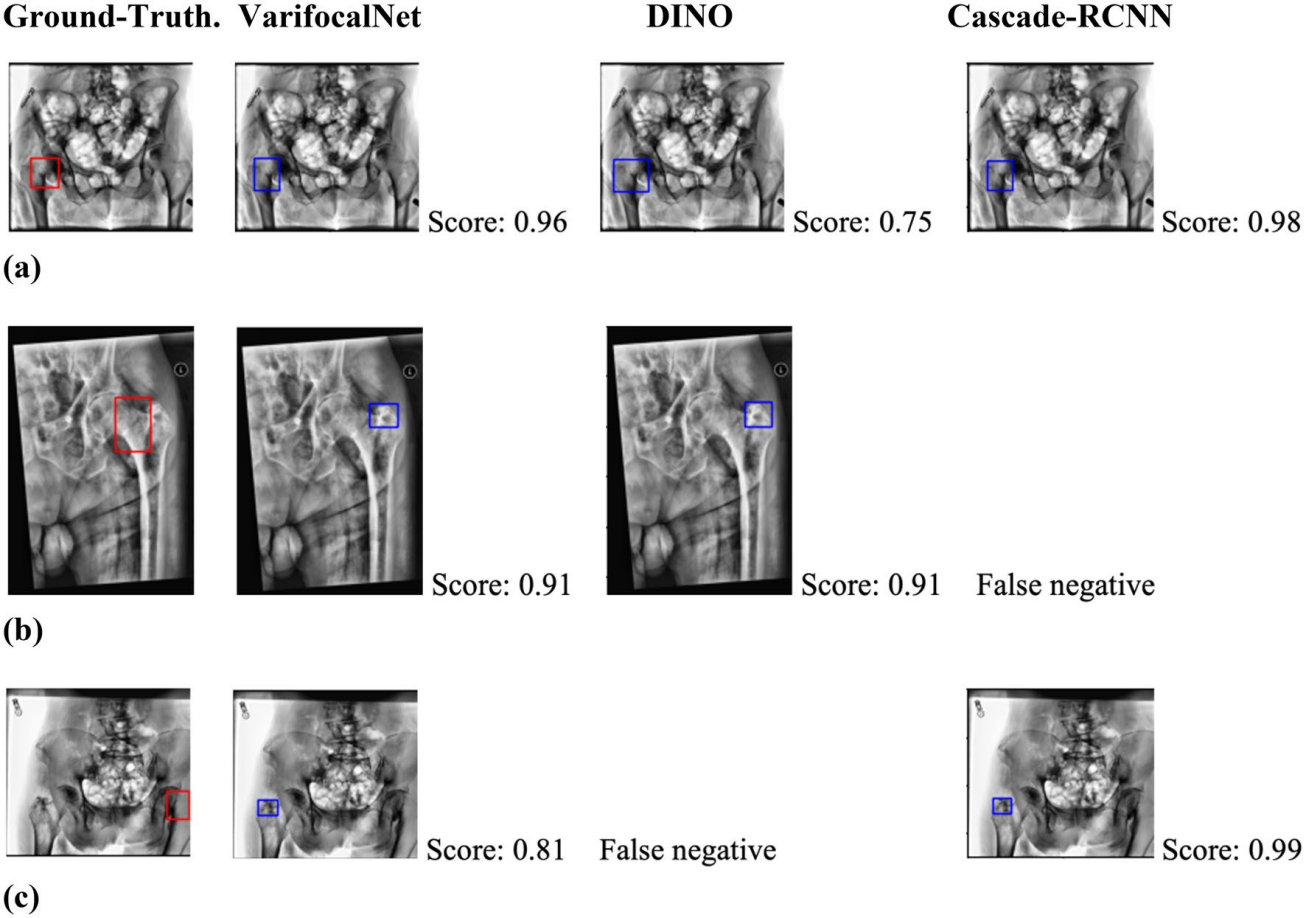
Qualitative examples of ground-truth fracture bounding boxes (left column) and VarifocalNet predictions (second column) against DINO (third column) and Cascade-RCNN (fourth column) predictions. The associated confidence score is provided on the right of each prediction image. Images are radiographs preprocessed via CLAHE, as described in Section "Data preprocessing".

Figure 3 compares fracture bounding box predictions of VarifocalNet against two competing methods with the highest average IOUs in Table 2: DINO and Cascade-RCNN. All three methods typically localized fracture regions of interest correctly compared to the corresponding ground-truths, demonstrated by the similar IOUs in Table 2 and exemplified by Fig. 3a. Figure 3b,c show the only two true positive predictions for which the VarifocalNet fracture box predictions did not overlap with ground-truth boxes. In both cases, DINO or Cascade-RCNN also made the same localization mistake or could not correctly classify the scan as positive for fracture. In particular, the scan in Fig. 3c shows the only scan for which VarifocalNet (as well as Cascade-RCNN) predicted the opposite side of the ground-truth as the fracture location. As this scan belonged to an 80-year-old female subject, we believe the contralateral side of the fractured hip introduced a challenge for both methods, given the systemic nature of fracture risk and the similarity of the two femurs^85–88^. Qualitative results confirmed that the fracture localization performance of VarifocalNet was on par with other competing methods, while also significantly improving fracture detection performance, as discussed in Section "Quantitative results".

**Figure 4:**
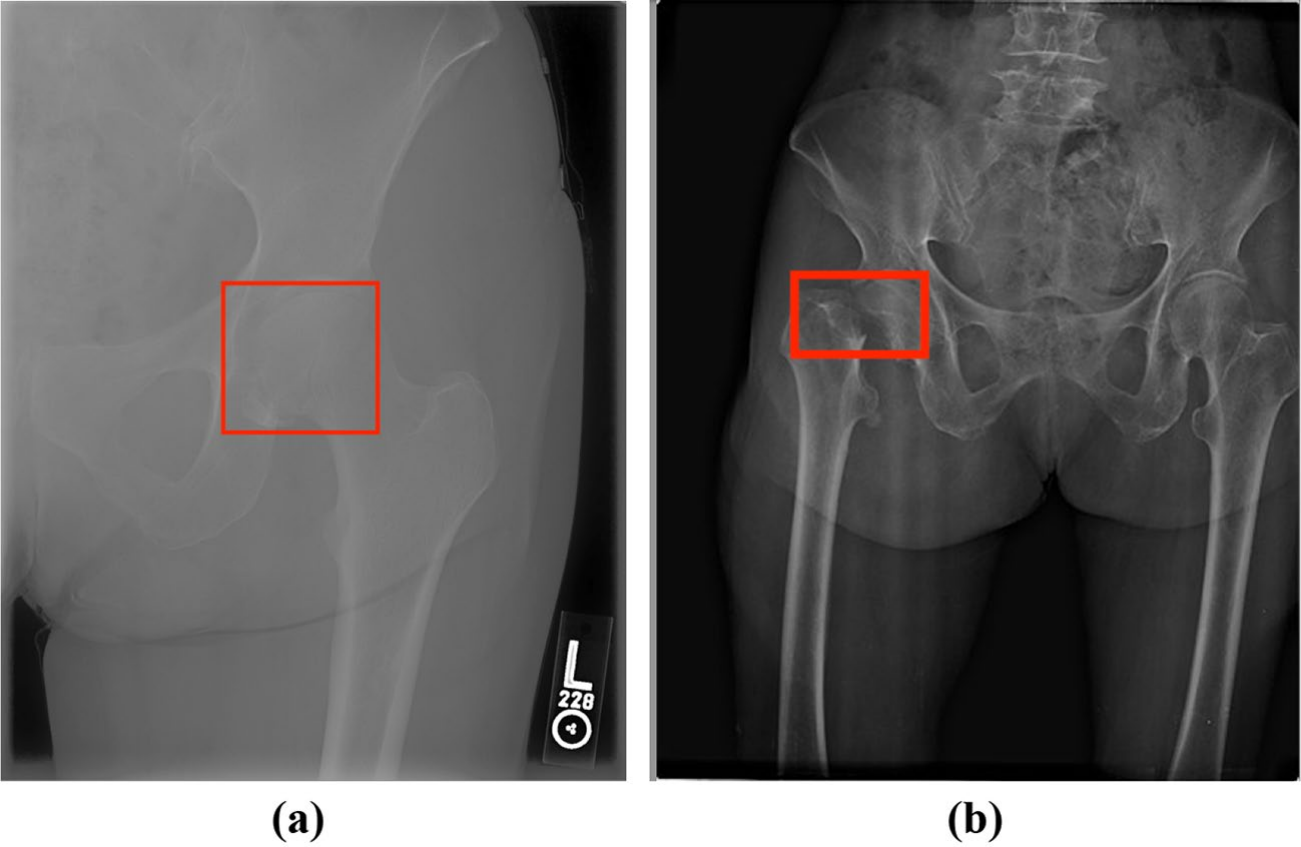
Ground-truth fracture scans falsely predicted as negative controls by VarifocalNet.

Figure 4 visualizes the only two ground-truth fracture scans falsely predicted as negative by VarifocalNet. As femoral head fractures are uncommon^89^ and represented by only 2% of the subjects in our dataset, Fig. 4a demonstrates a rare and difficult femoral head fracture scan for the proposed model. For the scan in Fig. 4b, VarifocalNet predicted a fracture bounding box with a confidence score falling slightly below the detection threshold. We believe the confidence score was lower since this scan was considerably more zoomed out of the hip region and contained most of the femur bone, compared to the other hip scans in Figs. 2 and 3.

### External validation

To assess the robustness and generalizability of the proposed method, we conducted further experiments on a publicly available dataset associated with two recent works^22,43^. The PelvixNet dataset^90^ comprised 100 frontal view plain radiographs of the hip with 50 scans collected from subjects with hip fractures and the remaining 50 from subjects without hip fractures. Included scans did not contain annotations of fracture locations. We used the models trained over our dataset to perform fracture detection on PelvixNet, with detection thresholds developed over our dataset as described in Section "Inference and evaluation metrics". Corresponding results are presented in Table 3.

**Table 3:** Comparison of VarifocalNet to competing methods over the external PelvixNet dataset.

Method		AUC	Specificity	Sensitivity	Accuracy	NPV	PPV
VarifocalNet	Metric	0.82	0.74	**0.78**	0.76	**0.77**	0.75
	CI ($\pm$)	0.08	0.09	0.08	0.08	0.08	0.08
DINO	Metric	**0.88**	**0.92**	0.46	0.69	0.63	**0.85**
	CI ($\pm$)	0.07	0.05	0.1	0.09	0.09	0.07
	p-value	10^–8^	10^–18^	10^–18^	10^–11^	10^–16^	10^–15^
FasterRCNN	Metric	0.83	0.86	0.68	**0.77**	0.73	0.83
	CI ($\pm$)	0.08	0.07	0.09	0.08	0.09	0.07
	p-value	0.8	10^–16^	10^–14^	0.15	10^–5^	10^–12^
CascadeRCNN	Metric	0.82	0.92	0.44	0.68	0.62	0.85
	CI( $\pm$)	0.08	0.05	0.1	0.09	0.1	0.07
	p-value	0.4	10^–18^	10^–18^	10^–13^	10^–16^	10^–15^
RetinaNet	Metric	0.83	0.76	0.74	0.75	0.75	0.76
	CI ($\pm$)	0.08	0.08	0.09	0.08	0.08	0.08
	p-value	0.8	0.58	10^–5^	0.28	0.02	0.36
FCOS	Metric	0.74	0.7	0.66	0.68	0.67	0.69
	CI ($\pm$)	0.01	0.09	0.09	0.09	0.09	0.09
	p-value	10^–12^	10^–7^	10^–16^	10^–13^	10^–13^	10^–9^
GCNet	Metric	0.79	0.9	0.44	0.67	0.62	0.81
	CI ($\pm$)	0.09	0.06	0.01	0.09	0.1	0.08
	p-value	10^–4^	10^–18^	10^–18^	10^–14^	10^–16^	10^–9^
DenseNet	Metric	0.83	0.82	0.52	0.67	0.63	0.74
	CI ($\pm$)	0.08	0.08	0.1	0.09	0.09	0.09
	p-value	0.8	10^–9^	10^–18^	10^–14^	10^–16^	0.2
EfficientNet	Metric	0.8	0.82	0.46	0.64	0.6	0.72
	CI ($\pm$)	0.09	0.08	0.1	0.09	0.1	0.09
	p-value	10^–3^	10^–9^	10^–18^	10^–17^	10^–17^	10^–4^

Fracture detection metrics include area under the receiver operating characteristic curve (AUC), sensitivity, specificity, accuracy, positive predictive value (PPV) and negative predictive value (NPV). For each metric, the row with the highest value is written in bold. Below each metric, the 95% confidence interval (CI) is written. For each method other than the proposed VarifocalNet, the p-values (for the two-sided Mann–Whitney nonparametric test) for comparing the metrics to those of VarifocalNet are also written.

VarifocalNet attained significantly higher sensitivity and NPV than other methods by up to 34% sensitivity (p-values < 10^–5^) and 17% NPV (p-values < 0.02), as well as the second highest accuracy that did not have a significant difference with the highest accuracy. Similar to the results over our dataset (c.f. Section "Quantitative results"), VarifocalNet further exhibited balance between sensitivity and specificity, while several other methods including DINO, DenseNet and EfficientNet resulted in severe imbalance by up to 48% difference between the two metrics. Moreover, end-to-end detection and localization models consistently outperformed DenseNet and EfficientNet, further underlining the benefit of localization in terms of robustness in detection performance. These results were also promising for potential applications in the clinical-care environment, where sensitivity is the most critical metric as false negatives can lead to delayed diagnosis or unrecognized fractures, while specificity should also be at a similar level in order to reduce unnecessary burden of time and cost for both clinicians and patients.

## Discussion

We employed and extended the state-of-the-art VarifocalNet^50^ for end-to-end proximal femur fracture detection and localization from plain radiography. Our retrospective dataset comprised 823 hip radiographs acquired from 150 fracture subjects and 362 non-fracture controls, with diverse patient parameters summarized in Table 1.

A large body of research has used deep learning models to identify or classify hip fractures from radiographs^20–32^, albeit lacking explicit localization of identified fractures. These approaches employed a plethora of well-established convolutional neural networks such as AlexNet^26^, GoogLeNet^26^, ResNet^29^, DenseNet^21,24,30–32^, EfficientNet^20^ and Xception^22,23^. Extensions included heatmap-based analysis via weighted class activation mapping (Grad-CAM)^20–23,29,31,32^, improved loss functions such as focal loss^30^, autoencoder networks for feature extraction^28^, and curriculum learning^25^. When trained over thousands of annotated hip radiographs, these detection models attained up to 0.99 AUC^29,31^. Our approach via VarifocalNet attained 0.98 AUC while using only 823 radiographs collected from 150 fracture subjects and 362 negative controls. Crucially, VarifocalNet performed joint detection and localization of proximal femur fractures, allowing the clinician to visualize and overread automated detection results to confirm or decide on further evaluation.

Several studies have detected and localized hip fractures from radiographs via deep learning, albeit requiring multiple cascaded models^33–37^. In particular, a neural network was first trained to zoom into the hip region on radiographs, using customized convolutional networks^33,34^ or well-established architectures such as AlexNet^35^ and Yolo^36^. A second network was then trained over the hip radiographs cropped around the hip to detect and classify fractures, with novel architectures including Siamese networks^37^ and vision transformers^36^. Developing and evaluating such cascaded approaches is less computationally efficient than end-to-end detection and localization approaches^38–40^. Furthermore, cascaded approaches may require manual data cleaning between cascaded models to address error propagation, as exemplified by Tanzi et al.^36^. Instead, our approach performed end-to-end detection and localization of proximal femur fractures via one deep-learning model based on VarifocalNet. More importantly, we tested a transformer model for the first time for end-to-end hip fracture detection and localization from plain radiography; Tanzi et al.^36^ instead used a transformer as the classification stage of a multi-stage cascaded model. VarifocalNet not only outperformed the state-of-the-art DINO transformer regarding clinical metrics but also took half the time on average to process a radiograph. Our results established that for small-sample settings like ours, FPNs remain state-of-the-art compared to transformer models requiring thousands of annotated images for training^36,84^.

Closer to our work, recent studies have performed end-to-end detection and localization of hip fractures from radiographs^41–47^. Jiménez-Sánchez et al.^41^ and Kazi et al.^42^ incorporated transformations (such as scaling and translation) into detection models, where all transformations were trained to maximize the detection performance. Unlike our work, these approaches did not use bounding box annotations of fracture and, accordingly, did not perform localization accurately^41^. Instead, most existing works used FPN models^43–47^, trained over fracture bounding box annotations for end-to-end detection and localization of hip fractures. FPNs tested for this task included Faster-RCNN^44^, Cascade-RCNN^75^, RetinaNet^76^, FCOS^77^, DCFPN^45^, ParallelNet^46^ and GCNet^47^. As presented in Section "Quantitative results", our study assessed FPNs based on clinically relevant metrics to establish the state-of-the-art. Our proposed model based on VarifocalNet outperformed Faster-RCNN, Cascade-RCNN, RetinaNet, FCOS and GCNet by up to 6% AUC, 14% sensitivity, 9% accuracy, and 12% NPV with p-values < 10^–4^. We did not evaluate DCFPN and ParallelNet, as they were outperformed by GCNet when tested over the same dataset^47^. Cheng et al.^43^ also proposed an FPN model, albeit requiring point annotations marking centers of fracture-related hip regions, rather than bounding box annotations that we considered. We focused on bounding box annotations due to the extensive literature with the same data annotation setting^33–36,43–46^, also noting that point annotations are typically used with other imaging modalities than radiography, such as histopathology^91–93^ and MRI^94^.

Our study has some limitations. While our dataset contained a similar number of radiographs of proximal femur fractures and negative controls (468 with fractures, 355 controls), samples with proximal femur fractures were collected from 150 subjects. This reduced the number of independent training and testing samples, further exacerbating small-sample challenges such as large confidence intervals in Table 2. Another challenge was the imbalance of genders in our dataset, containing twice the number of female subjects than male subjects with proximal femur fractures. This resulted in a higher AUC of fracture detection over female subjects than males, as they were better represented in training. While this imbalance agreed with the literature on hip fractures^14,53^, collecting more scans from male subjects to augment our dataset would improve performance generalization. Moreover, we believe that the performance gap between our dataset and PelvixNet by all models may be due to the fact that our dataset mainly focused on proximal femur fractures due to bone fragility, while PelvixNet mainly included fractures due to trauma. Including other fracture types such as trauma and pathologies other than osteoporosis would further improve generalization.

## Conclusion

We evaluated deep learning models on end-to-end proximal femur fracture detection and localization from plain radiography with clinically relevant metrics, focusing on the state-of-the-art VarifocalNet FPN. Tested over 823 hip radiographs of 150 fracture subjects and 362 controls, VarifocalNet attained 0.94 specificity and 0.95 sensitivity, outperforming five benchmark FPNs. Taking the first steps in implementing a transformer model for our task, VarifocalNet further outperformed the transformer network DINO and confirmed FPNs as state-of-the-art for small-sample settings such as ours. Employing a highly sensitive and specific automated detection model for proximal femur fracture detection can aid experts in accurate diagnosis. This can reduce further advanced imaging requirements such as CT and MRI, saving patients and healthcare facilities time and resources. Our study focused on highly accurate detection of proximal femur fractures from radiographs but did not currently incorporate classification of fracture types^36^ or grades^33^. Collecting such annotations and extending VarifocalNet for classification and localization of proximal femur fractures of diverse types is an open direction.

### Supplementary Information

Supplementary Tables.

## Data Availability

The datasets generated and analyzed during the current study are not publicly available due to being supported by an NIH SBIR grant award. As outlined in the 2023 NIH Data Management and Sharing Policy, “SBIR and Small Business Technology Transfer (STTR) recipients may retain the rights to data generated during the performance of an SBIR or STTR award for up to 20 years after the award date, per the SBIR and STTR Program Policy Directive but are available on reasonable request from the corresponding author."
