# Supplementary material for: Proximal femur fracture detection on plain radiography via feature pyramid networks
Source: Sci Rep. 2024 May 27;14:12046. doi: 10.1038/s41598-024-63001-2 (PMC11130146; doi:10.1038/s41598-024-63001-2)
Supplement: Supplementary file 1 — Supplementary Tables. [file 41598_2024_63001_MOESM1_ESM.pdf]

Proximal Femur Fracture Detection on Plain Radiography via Feature Pyramid Networks

Supplementary Material

İlkay Yıldız Potter, PhD [a *]; Diana Yeritsyan, BS [b, c]; Sarah Mahar, BS, MPH [b, c]; Nadim Kheir, MD [b, c]; Aidin Vaziri, MS [a]; Melissa Putman, MD [d]; Edward K. Rodriguez, MD, PhD [b, c]; Jim Wu, MD [e]; Ara Nazarian, PhD [b, c, f †]; Ashkan Vaziri, PhD [a †]

[a] BioSensics, LLC, 57 Chapel Street, Newton, MA 02458, United States
[b] Carl J. Shapiro Department of Orthopaedic Surgery, Beth Israel Deaconess Medical Center (BIDMC) and Harvard Medical School, 330 Brookline Avenue, Stoneman 10, Boston, MA 02215, United States
[c] Musculoskeletal Translational Innovation Initiative, Beth Israel Deaconess Medical Center and Harvard Medical School, 330 Brookline Avenue RN123, Boston, MA 02215, United States
[d] Division of Endocrinology, Massachusetts General Hospital and Harvard Medical School, 55 Fruit Street, Boston, MA 02114
[e] Department of Radiology, Massachusetts General Brigham (MGB) and Harvard Medical School, 75 Francis Street, Boston, MA 02215, United States
[f] Department of Orthopaedic Surgery, Yerevan State University, Yerevan, Armenia

[*] Corresponding author: ilkay.yildiz@biosensics.com
[†] These authors have contributed equally to this work as senior authors.

Table S.1: Scan level age, gender and proximal femur fracture presence distribution

	Female		Male		Unknown		Sum
**Age**	**Fracture**	**Negative Control**	**Fracture**	**Negative Control**	**Fracture**	**Negative Control**	
18-30	1	11	0	12	0	0	24
30-40	0	7	0	9	0	0	16
40-50	0	6	4	12	0	0	22
50-60	2	14	17	20	0	0	53
60-70	39	19	17	15	0	0	90
70-80	93	16	38	14	0	0	161
80-90	127	16	32	8	0	0	183
90-100	61	10	9	5	0	0	85
Unknown	0	0	0	0	28	161	189
**Sum**	323	99	117	95	28	161	823

Table S.2: Scan level radiography imaging device distribution

	**Fracture (n=468)**	**Negative Control (n=355)**
**Device, n (%)**		
General Electric (GE) Thunder	120 (26%)	88 (24%)
GE Centricity	2 (1%)	0
GE Revolution XR/d	3 (1%)	2 (1%)
Kodak Carestream DRX-1	5 (1%)	6 (2%)
Kodak Carestream DRX-Evolution	80 (16%)	41 (10%)
Fuji Photo film	93 (20%)	20 (6%)
Philips Digital Diagnostic	18 (4%)	18 (5%)
Unknown	147 (31%)	180 (52%)

Table S.3: Detailed breakdown of the base neural network architecture ResNeXt-101 in VarifocalNet, comprising convolutional (Conv), batch normalization (BatchNorm), ReLU activation, max pooling (MaxPool) and deformable convolution (DeformConv) layers. Residual Block (x *k*) implies *k* times repetition of the layers in the cell, with residual connections between the input and output. For each Conv and DeformConv, first two elements represent the number of input and output channels, respectively. For each BatchNorm, the first element represents the number of channels.

Conv: 3, 64, kernel_size=(7, 7), stride=(2, 2), padding=(3,3), bias=False
BatchNorm: 64, momentum=0.1
ReLU
MaxPool: kernel_size=3, stride=2, padding=1, dilation=1
Conv: 64, 256, kernel_size=(1, 1), stride=(1, 1)
<u>Residual Block (x3)</u><br>Conv: 256, 256, kernel_size=(1, 1), stride=(1, 1)<br>BatchNorm: 256, momentum=0.1, affine=True,<br>Conv: 256, 256, kernel_size=(3, 3), stride=(1, 1), padding=(1, 1), groups=64, bias=False<br>BatchNorm: 256, momentum=0.1, affine=True,<br>Conv: 256, 256, kernel_size=(1, 1), stride=(1, 1), bias=False<br>BatchNorm: 256, momentum=0.1, affine=True<br>ReLU
Conv: 256, 512, kernel_size=(1, 1), stride=(1, 1)
<u>Residual Block (x4)</u><br>BatchNorm: 512, momentum=0.1, affine=True<br>DeformConv: 512, 27, kernel_size=(3, 3), stride=(2, 2), padding=(1, 1)<br>BatchNorm: 512, momentum=0.1, affine=True,<br>Conv: 512, 512, kernel_size=(1, 1), stride=(1, 1), bias=False<br>BatchNorm: 512, momentum=0.1, affine=True<br>ReLU
Conv: 512, 1024, kernel_size=(1, 1), stride=(1, 1)
<u>Residual Block (x23)</u><br>BatchNorm: 1024, momentum=0.1, affine=True<br>DeformConv: 1024, 27, kernel_size=(3, 3), stride=(2, 2), padding=(1, 1)<br>BatchNorm: 1024, momentum=0.1, affine=True<br>Conv: 1024, 1024, kernel_size=(1, 1), stride=(1, 1), bias=False<br>BatchNorm: 1024, momentum=0.1, affine=True<br>ReLU
Conv: 1024, 2048, kernel_size=(1, 1), stride=(1, 1)

Residual Block (x3)<br>BatchNorm: 2048, momentum=0.1, affine=True<br>DeformConv: 2048, 27, kernel_size=(3, 3), stride=(2, 2), padding=(1, 1)<br>BatchNorm: 2048, momentum=0.1, affine=True<br>Conv: 2048, 2048, kernel_size=(1, 1), stride=(1, 1), bias=False<br>BatchNorm: 2048, momentum=0.1, affine=True<br>ReLU

Table S.4: Detailed breakdown of feature pyramid architecture in VarifocalNet. Each lateral connection merges feature maps of the same spatial size between the lower resolution and higher resolution pathways in the pyramid. For each convolutional layer (Conv), first two elements represent the number of input and output channels, respectively.

Lateral Connections	Conv: 512, 256, kernel_size=(1, 1), stride=(1, 1)
	Conv: 1024, 256, kernel_size=(1, 1), stride=(1, 1)
	Conv: 2048, 256, kernel_size=(1, 1), stride=(1, 1)
Pyramid Layers	Conv: 256, 256, kernel_size=(3, 3), stride=(1, 1), padding=(1, 1)
	Conv: 256, 256, kernel_size=(3, 3), stride=(1, 1), padding=(1, 1)
	Conv: 256, 256, kernel_size=(3, 3), stride=(1, 1), padding=(1, 1)
	Conv: 256, 256, kernel_size=(3, 3), stride=(1, 1), padding=(1, 1)
	Conv: 256, 256, kernel_size=(3, 3), stride=(1, 1), padding=(1, 1)

Table S.5: Detailed breakdown of bounding box and confidence score prediction architecture in VarifocalNet, comprising convolutional (Conv), group normalization (GroupNorm), ReLU activation and deformable convolution (DeformConv) layers. For each Conv, GroupNorm and DeformConv, first two elements represent the number of input and output channels, respectively. Both bounding box and confidence score prediction stages are used repeatedly for the five different resolution outputs from the feature pyramid.

Bounding box	Conv: 256, 256, kernel_size=(3, 3), stride=(1, 1), padding=(1, 1), bias=False
	GroupNorm: 32, 256, affine=True
	ReLU
	DeformConv: 256, 256, kernel_size=(3, 3), stride=(1, 1), padding=(1, 1), dilation=(1, 1), groups=1, bias=False
	Conv: 256, 4, kernel_size=(3, 3), stride=(1, 1), padding=(1, 1)
Confidence score	DeformConv: 256, 256, kernel_size=(3, 3), stride=(1, 1), padding=(1, 1), dilation=(1, 1), groups=1, bias=False
	Conv: 256, 1, kernel_size=(3, 3), stride=(1, 1), padding=(1, 1)
